# Cost containment by peer prior authorization program for second line treatment in patients with retinal disease

**DOI:** 10.1186/s13584-021-00437-1

**Published:** 2021-01-25

**Authors:** Amir Rosenblatt, Igal Hekselman, Irit Rosenblatt, Idan Hekselman, Dan Gaton

**Affiliations:** 1 Division of Ophthalmology, Tel Aviv Sourasky Medical Center (Ichilov), 6 Weizmann Street, Tel Aviv, Israel; 2 Sackler Faculty of Medicine, Tel Aviv University, Tel Aviv, Israel; 3 Clalit Mushlam Health Insurance Systems, Clalit Health Services, Ramat Gan, Israel; 4 Department of Ophthalmology, Beilinson and Hasharon, Rabin Medical Center, Petah-Tikva, Israel; 5 Medical School for International Health, Ben-Gurion University of the Negev, Be’er Sheva, Israel

**Keywords:** Cost-containment, Retinal disease, Gatekeeping, Sentinel, Ethical dilemma, Ranibizumab, Bevacizumab, Health maintenance organization

## Abstract

**Background:**

High and increasing drug prices have prompted the establishment of a broad range of cost-containment treatment policies in health systems globally. In 2012, the supplemental insurance program of a large Israeli health maintenance organization (Clalit Health Services) introduced a prior authorization process for second-line use of ranibizumab in patients with retinal disease for whom treatment with bevacizumab proved to be ineffective. A Clalit steering committee established authorization criteria based on cost and periodically updated clinical considerations, while a team of ophthalmic specialists evaluated their colleagues’ individual patient subsidization requests, based on the funding criteria. The objectives of this study were to detail this unique authorization process and study its effectiveness in limiting unwarranted spending, while allowing for a smooth transition to a second-line more expensive drug when needed.

**Methods:**

A retrospective cohort study including all applications for a first or ongoing treatment with ranibizumab, for one or both eyes, received during March 1, 2012 - December 31, 2015. The key parameters examined were percentages of requests from patients treated by first line treatment bevacizumab, requests approved, reapplications, and results. Requests studied include reapplications and requests for treatment continuation.

**Results:**

During the study period, Clalit affiliated ophthalmologists’ submitted 16,778 funding applications for intravitreal ranibizumab treatment on behalf of 5642 patients who applied for approximately three applications. An efficient sentinel effect was achieved, resulting in only 31% of patients treated with bevacizumab applying for treatment, while maintaining extremely high accessibility to second line treatment with almost 95% of requests being approved.

**Conclusions:**

The data presented shows a low request rate for funding with a high approval rate, proving this peer reviewed report-based authorization process successfully achieved a sentinel effect while controlling cost. We suggest this innovative model be considered in similar decisions processes.

## Background

Rising drug costs and the aging population in high-income countries pose cost-containment challenges to healthcare systems [1]. Increasing patient co-payments for more expensive drugs can lead to the decreased or discontinued use of essential medications [2]. An alternative solution is the formulation of prior-authorization policies for specific drugs, which have been shown to increase economic savings in health maintenance organizations (HMOs) by reducing expensive and unnecessary drug prescriptions [3, 4].

Visual impairment and blindness pose a serious medical, social, and economic burden [5]. Prompted by studies showing that anti-vascular endothelial growth factor (anti-VEGF) slows the progression of vision loss in patients with various retinal diseases [6–10], Genentech Inc. (South San Francisco, USA) developed two anti-VEGF drugs: bevacizumab (Avastin®), marketed by Roche (Basel, Switzerland), and ranibizumab (Lucentis®), marketed by Novartis (Basel, Switzerland). Only the more expensive ranibizumab was approved by the U.S. Food and Drug Administration (FDA) and European Commission for intra ocular use. However, both drugs were found similarly effective and safe for the treatment of age-related macular degeneration (AMD) [11–16] and diabetic retinopathy [17]. Therefore, a policy directed at increasing the first-line use of bevacizumab was put in place in Israel and other countries. Today, the off-label first-line use of bevacizumab is growing worldwide. According to one study from the U.S., off-label drug use in general accounts for 21% of all commonly prescribed medications [18]. However, some patients may have limited to no response to initial anti-VEGF treatment, while others lose the therapeutic effect during the long-term treatment course [19]. One strategy to treat such patients is by switching to a different agent. Changing from one anti-VEGF drug to another has been shown to benefit certain patients and is a viable treatment option for non-responders [20]. In the case of ranibizumab, it was shown as effective treatment for patients with diabetic macular edema (DME) or AMD who failed to respond to bevacizumab [7, 10, 21–23].

In Israel, the National Health Insurance Law provides universal, high-quality, government-subsidized coverage of approved medical services, drugs, and equipment, denoted the health “basket” for all citizens and residents [24]. The basket’s contents are reviewed annually by a dedicated committee of healthcare professionals and public figures. Addition of novel treatments and technologies is determined based on expected patient benefits, patient load, and projected costs. Individual medical services are allocated by four national nonprofit HMOs. Membership in any HMO is mandatory. Members pay a minimum health tax and select an HMO with its basic basket services, and may also purchase supplementary health plans through their HMO or in the private sector.

Clalit Health Services is the largest HMO in Israel (and second largest in the world), insuring more than 53% of the population (approximately 4.3 million members as of 2014). About 70% of Clalit Health Services members owned its supplementary health plan, “Clalit Mushlam”, in the beginning of this study. Treatments not included in the basket that are covered by Clalit Mushlam, are determined by a steering committee of medical professionals, health administrators, and health economists. Final decisions are based on estimated clinical and financial data. Applications for out-of-the-basket treatments for individual patients are submitted by treating physicians. These are sent for revision to select physicians, who decide if to provide subsidization, according to the criteria determined by Clalit Mushlam’s steering committee.

In 2008, Clalit Health Services added bevacizumab for off-label treatment of retinal diseases to its list of medications offered almost free-of-charge to members. The indications for bevacizumab were AMD, DME, and central and branch retinal vein occlusions (CRVO and BRVO, respectively). In March 2012, ranibizumab was authorized for use on the HMO’s supplementary plan, Clalit Mushlam, for the identical indications of bevacizumab, in case of retinal diseases. Expected costs were assessed in advance, taking into account co-payments of $100 by the patient. Notably, one injection of ranibizumab in the Israeli private sector costs about $1600, which is more than the monthly pension of most retirees, the largest potential population expected to need ranibizumab. Insufficient response to bevacizumab treatment, in one or both eyes, led the treating ophthalmologist to request subsidization for ranibizumab from the HMO.

While reviewing possibilities of how to regulate access to new and expensive drugs, the scope of options available for an HMO lies on a scale between two extremities. The first, is funding a treatment for all patients (i.e., complete coverage) allowing maximal accessibility to health care and maximum physician control, but at a high cost. The second, not funding the treatment altogether (i.e., no coverage) allows for minimal expenditure and maximum HMO control, but with minimal accessibility to newer developed drugs.

In order to provide good accessibility to health while maintaining a responsible budget, a compromise between the two extreme options is found by either limiting per patient expenditure (by applying a treatment quota for each patient or implementing a very high co-pay), or by limiting patient’s access (through strict clinical criteria and/or a prior authorization process).

To examine the possibility of coverage for ranibizumab by Clalit Mushlam, a steering committee was established in 2012. The aim of the committee was to allow funding and access to second-line treatment for those needing it while building a sustainable economically responsible policy. The process had to maintain trust and good working relations between physicians and the HMO.

The committee was comprised of four medical professionals (three leading ophthalmologists and a leading neurologist), three health administrators (two medical doctors and a pharmacist), and three health economists. The proposed model for the treatment was to use ranibizumab as a second-line treatment, set definitions and treatment criteria, and additionally to maintain trust between physicians and the HMO, with no reading center involved in regulating, without tracking prescription rates of specific physicians and all decisions based on reports by the treating physicians. The steering committee established treatment and funding protocols for retinal diseases, including AMD, DME and CRVO/BRVO. Later in February 2014, choroidal neovascularization in pathologic myopia was added to the indications list, according to evidence-based knowledge accumulated in the literature and Micromedex database.

The objectives of this study were to describe this novel approach for a peer-examination process of ranibizumab treatment recommendation in retinal diseases and to study its effectiveness in limiting unwarranted spending, while allowing for a smooth transition to a second-line, more expensive drug when needed.

## Methods

The study was approved by the Clalit institutional community review board.

The study cohort included all the applications from Clalit Mushlam members from March 1, 2012 to December 31, 2015 for a first or ongoing treatment with ranibizumab, for one or both eyes.

### Indications of prior authorization

The steering committee established treatment and funding protocols for retinal diseases, including AMD, DME, and CRVO/BRVO. Later in February 2014, choroidal neovascularization in pathologic myopia was added to the indications list, based on evidence-based knowledge accumulated in the literature and Micromedex database.

### Criteria for prior authorization

Bevacizumab was designated the first-line treatment.

Candidates for switching to ranibizumab treatment were defined as members of Clalit Mushlam, who failed to respond to bevacizumab after *at least* three consecutive injections (in the same eye), with 4–6 weeks intervals between injections during the last 6 months, administered in a public hospital. Bevacizumab failure was defined as at least one of the followings: (i) optical coherence tomography (OCT) findings of increased central retinal thickness in addition to presence of fluids; (ii) disease-related decrease in visual acuity (VA) by more than one line; (iii) decreased central retinal thickness of < 50 μm or < 10% in the presence of edema; and (iv) toxic or inflammatory reaction to bevacizumab.

In patients previously given ranibizumab, the treating ophthalmologist could request its continuation in cases where ranibizumab was the last drug administered and a good response reported after three consecutive injections. A good response was determined as (i) improvement in central retinal thickness of > 50 μm or > 10% according to OCT; (ii) improvement in VA due to improvement in retinal fluids after first three ranibizumab consecutive injections; or (iii) stabilization of these parameters after more than two ranibizumab treatment series.

In addition to the numerical information of VA and OCT findings, a general assessment of improvement, stability, or deterioration is required. Additionally, physicians could add reasoning or explanations for consideration.

Applications for ranibizumab treatment funding were rejected where there was lack of improvement during ranibizumab treatment, missing patient data (injection dates, visual testing, and OCT findings before and after treatment with ranibizumab), toxic or inflammatory reaction to ranibizumab, ocular or systemic adverse events, or diagnosis other than those approved.

Patients who did not meet these criteria according to submitted data, or who failed to respond to ranibizumab treatment in the past, were rejected for ranibizumab funding. After rejection, treating ophthalmologists were permitted to re-apply for their patients using a revised application with additional explanations.

### Process of requests submission for ranibizumab funding

**Fig. 1:**
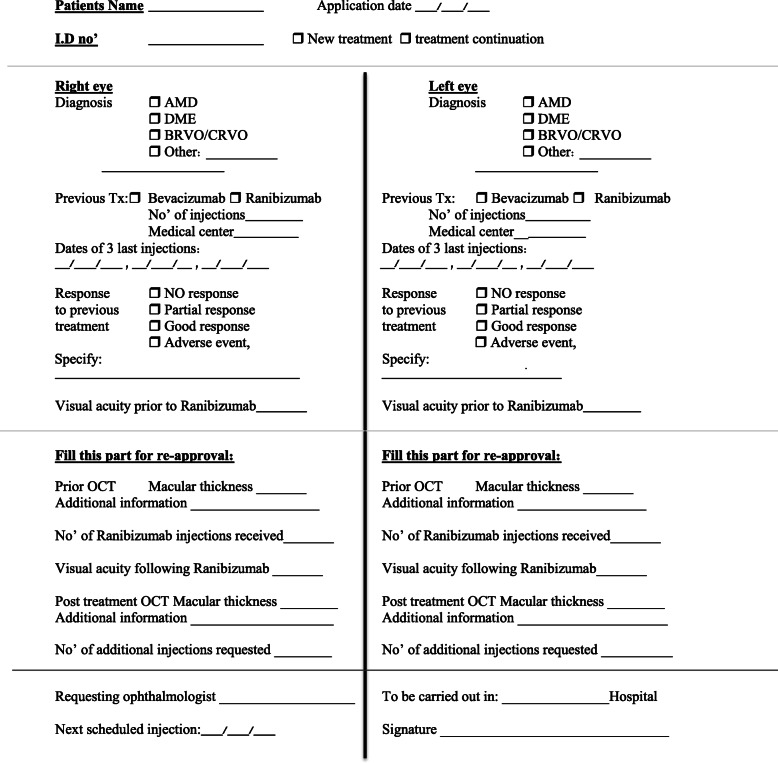
Request form. Clalit Mushlam application form for second line Ranibizumab treatment funding (source: authors’ translation of Clalit Mushlam application form

We phrased an approval application form for the request of ranibizumab treatment funding (Fig. 1). This application was submitted to the ophthalmic specialists team by the treating ophthalmologists employed at any of the 20 public hospital-affiliated retina units in Israel that treat individuals insured by Clalit Mushlam. Although usually not needed, supplementary medical records about the patients were sent directly from the retina units or the primary clinic in the community.

**Fig. 2:**
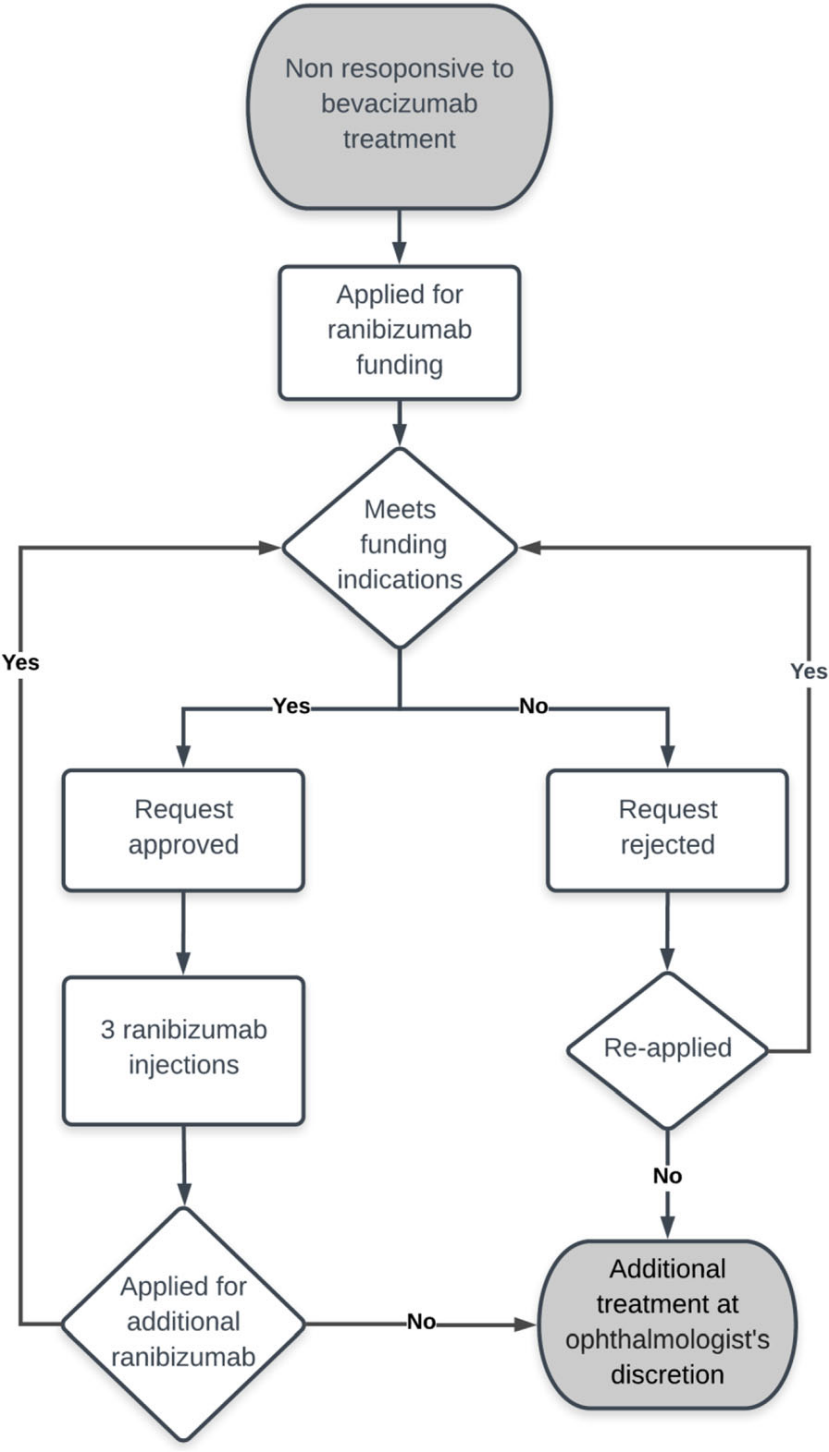
Funding process flowchart. Ranibizumab funding request process flowchart (source: authors’ visual representation of Clalit Mushlams’ algorithm for ranibizumab funding process)

When all the required data were submitted, the form was forwarded to the ophthalmic specialists’ team for evaluation by its representatives. Currently the team consists of two specialists working independently on an internet-based platform, who use the application forms’ information to assess the effectiveness of treatment to date, as well as the need to start, continue, or renew ranibizumab administration**.** Notably, neither the ophthalmic specialist team nor the treating ophthalmologists requesting the funding were provided with information regarding the financial considerations of the steering committee, or expenses of Clalit Mushlam. The patient, primary care unit personnel, and retina unit personnel were all blinded to the identity of the ophthalmic specialists who examined the application. The responses to the applications were sent via email, along with explanations, if necessary, to the patient’s primary care physician and retina unit treating ophthalmologist who submitted the application form (Fig. 2). A single funding approval covered three injection administrations and expired after a 6-month period. The co-payment for patients prescribed ranibizumab, determined according to the regulations of Clalit Mushlam, was about $100 per injection (drug and its administration’s costs).

In patients for whom funding for the treatment with ranibizumab was denied, the treating ophthalmologists could either continue with bevacizumab injections, stop treatment, or send an amended application form for re-evaluation of the request, if additional data were available (Fig. 2).

### Data collection

All data from applications submitted to Clalit Mushlam are recorded in an electronic medical form and forwarded to the authorization committee, as described above. For purposes of this study, we collected the basic demographic details of the applicants, their application history, and all information provided on the application forms. This including prior bevacizumab injection dates, prior ranibizumab injection dates, VA and central macular thickness (CMT) of both baseline and recent visits, and history of prior requests and their outcome. Applications missing data relevant for documentation were excluded. The data was uploaded to an electronic file in encrypted form to conceal patient identity. Descriptive summaries and statistical analyses were generated using Python 2.7.12 language via Scikit-learn library.

### Outcome measures

Overall number of requests approved and number of injections needed was assessed. As the payment from the HMO to medical facilities for administering the injection is comparable between all drugs overall costs and savings depend only on number of second line injections administered.

The process of requests starts by the Retina Specialist who files a request filling in data taken from the EMR of his patient. This procedure takes Less than 5 min to find the needed data and enter it to the request form. It is than scanned to an electronic request file. This is considered part of the job requirements for each medical staff in retina clinics in all public hospitals, thus, carries no extra cost.

### Process measures

This paper will assess the compliance of front-line physicians with guidelines assessed by and extent of rejected request and overall approval rate, to ensure patients are not prevented justified access to healthcare.

## Results

### Yield of the ranibizumab approval process

According to an external report supplied by Clalit Health Services prior to the study period, approximately 22,000 individuals were treated for retinal diseases with bevacizumab in 2011. By their estimation, they received a total of approximately 200,000 injections annually.

Approximately 18,000 (82%) bevacizumab-treated patients had supplementary Clalit Mushlam insurance, accounting for 164,000 bevacizumab injections. The steering committee estimated the consumption of nearly 16,000 vials of ranibizumab annually, based on: (i) number of patients treated with bevacizumab; (ii) expected compliance of treating ophthalmologists and ophthalmic experts to the guidelines; (iii) patients’ adherence to ophthalmologists’ recommendations; (iv) accepted treatment protocols for ranibizumab by diagnosis; and (v) expected learning curve of the funding process by the treating ophthalmologists.

After the establishment of the request submission process for ranibizumab funding, Clalit Mushlam announced it to the managers of all retina units in all public hospitals. Our study included 5642 patients (31% of bevacizumab-treated members of Clalit Mushlam) who applied for second-line ranibizumab treatment by a treating ophthalmologist in a public hospital-affiliated retina unit.

**Fig. 3:**
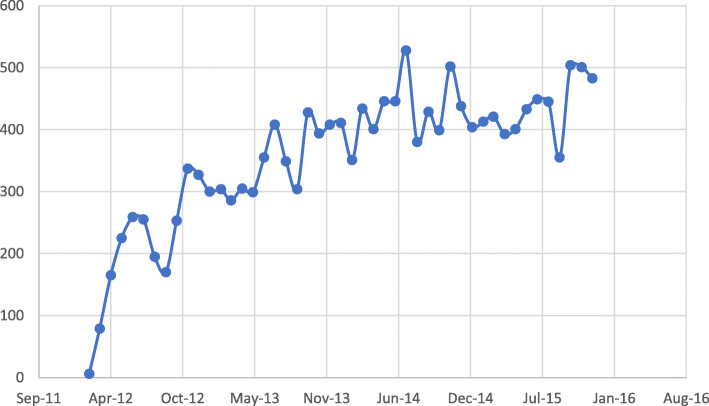
Monthly requests submission rate (source: authors’ analysis of data from Clalit Mushlams’ cohort of members funding applications, March 1, 2012 to December 31, 2015)

These patients included 2867 (51%) females and 2755 (49%) males, with a mean age of 75.3 ± 11.13 years (Table 1). A total of 16,778 applications for the initiation or continuation of ranibizumab treatment were submitted for evaluation to the ophthalmic specialists team. The rate of application submission was 2.97 applications per patient. The request submission rate was few requests at the beginning of the study, elevated up to 528 requests per month, and its mean was 357 requests per month throughout the study period (Fig. 3). The entire process, from submission of the application form to either approval or rejection of the request took up to 72 h.

**Table 1:** Inquiries and approvals for Ranibizumab according to the type of retinal disease, age and gender (source: authors’ analysis of data from Clalit Mushlams’ cohort of members funding applications, March 1, 2012 to December 31, 2015]

	AMD	Other CNV	RVO	DME	Other	Complex Dx	Total
	*n* = 3445	*n* = 16	*n* = 467	*n* = 1365	*n* = 25	*n* = 324	*n* = 5642
Age - years, mean ± SD	79.7 ± 9.02	71.6 ± 11.02	61.1 ± 15.61	66.7 ± 9.12	71.1 ± 12.29	70.0 ± 7.73	75.3 ± 11.13
Gender-female, n(%)	1919 (56%)	8 (50%)	234 (50%)	560 (41%)	12 (48%)	134 (41%)	2867 (51%)
Total Inquiries, n	11,107	24	1054	3053	31	1509	16,778
Approved inquiries, n(%)	8700 (78%)	19 (79%)	900 (85%)	2575 (84%)	0 (0%)	1084 (72%)	13,278 (79%)

*SD* standard deviation, *n* number of eyes, *Age* Age at examination, *AMD* neovascular age related macular degeneration, *CNV* choroidal neovascularization, *RVO* retinal vein occlusion, *DME* diabetic macular edema, *Dx* diagnosis

### Requests analysis and approval rates

Analysis revealed high approval rates of the funding requests. As many as 80.2% of requests were approved, after submitting the first application form (Table 2). Specifically, 84% of first-time requests and 76% of continuation requests for ranibizumab treatment were approved after the first application. Moreover, even after rejection, 14% of all requests were approved after resubmissions of the amended application form. Eventually, only 808 (5.7%) requests were rejected. Out of these, 288 requests (5.1% out of first-time requests) were rejected, while 520 continuation requests were denied, leading to termination of these patients’ treatment funding. Ultimately, 13,278 requests for subsidization were approved (average of 2.35 per patient).

**Table 2:** Summary of patients’ requests for funding of Ranibizumab (source: authors’ analysis of data from Clalit Mushlams’ cohort of members funding applications, March 1, 2012 to December 31, 2015)

Previously approved Tx	Pt applying	Pt dropped out from previous Tx	Total No′ of applications	Mean No′ of applications per Pt	Pt approved w/o further scrutiny	Pt approved after repeated application	Pt not receiving funding	Total Pt approved for funding
					N (%)	N (%)	N (%)	N (%)
0	5642	–	6452	1.1	4740 (84.0%)	614 (10.9%)	288 (5.1%)	5354 (94.9%)
1	3325	2029	4143	1.2	2519 (75.8%)	588 (17.7%)	218 (6.5%)	3107 (93.5%)
2	2005	1102	2454	1.2	1556 (77.6%)	321 (16.0%)	128 (6.4%)	1877 (93.6%)
3	1233	644	1478	1.2	986 (80.0%)	184 (14.9%)	63 (5.1%)	1170 (94.9%)
4	791	379	963	1.2	631 (79.8%)	121 (15.3%)	39 (4.9%)	752 (95.1%)
5	478	274	560	1.2	393 (82.2%)	58 (12.1%)	27 (5/7%)	451 (94.3%)
6	273	178	329	1.2	208 (76.2%)	42 (15.4%)	23 (8.4%)	250 (91.6%)
7	160	90	191	1.2	126 (78.8%)	23 (14.4%)	11 (6.8%)	149 (93.2%)
8	86	63	107	1.2	63 (73.3%)	15 (17.4%)	8 (9.3%)	78 (90.7%)
9	46	32	48	1	44 (95.7%)	2 (4.3%)	0 (0%)	46 (100.0%)
10	22	24	24	1.1	19 (86.4%)	2 (9.1%)	1 (4.5%)	21 (95.5%)
11	12	9	14	1.2	9 (75.0%)	2 (16.7%)	1 (8.3%)	11 (91.7%)
12	6	5	7	1.2	4 (66.7%)	1 (16.7%)	1 (6.6%)	5 (83.4%)
13	3	2	4	1.3	2 (66.7%)	1 (33.3%)	0 (0%)	3 (100.0%)
14	2	1	2	1	2 (100.0%)	0 (0%)	0 (0%)	2 (100.0%)
15	1	1	1	1	1 (100.0%)	0 (0%)	0 (0%)	1 (100.0%)
16	1	0	1	1	1 (100.0%)	1 (100.0%)	0 (0%)	1 (100.0%)
Total	14,086	4833	16,778	3	11,303 (80.2%)	1975 (14.02%)	808 (5.7%)	13,278 (94.3%)

*N* number of patients, *Pt* patients, *Tx* treatment

De facto, during the study period nearly 99.8% of approved requests led to a series of three ranibizumab injections. According to a report of Clalit Health Services, as many as 41,739 ranibizumab injections were supplied to the retina units.

A diagnosis-based analysis reveals that the most frequent indication for treatment was AMD (61%), followed by DME (24%) and the rest had a complex diagnosis, CRVO/BRVO or miscellaneous (Table 1). The approval rate for requests differed significantly by indication (*P* < 0.001): 85, 88, 87, 0% for AMD, DME, CRVO/BRVO and miscellaneous diagnoses, respectively. We further excluded patients with miscellaneous diagnoses, due to inadequacy to relevant indications. However, the likelihood of approval was lower for patients with AMD (OR 0.91, 95% CI 0.88–0.93) than for patients with DME (OR 1.27, 95% CI 1.77–1.37) or CRVO (OR 1.39, 95% CI 1.20–1.59), when compared to the other diagnoses.

Overall, this process enabled treating approximately 18,000 patients with retinal diseases with the physician’s medication of choice. While most of the patients received the less expensive, but not less effective treatment, only a reduced number of non-responders were determined as appropriate for funding with almost a 10-fold costlier treatment.

## Discussion

Ideally, comprehensive health coverage should be affordable, accessible, and appropriate [25, 26]. Here, we described a prior authorization program intended to effectively allocate a costly treatment, while staying within a budgetary framework. A steering committee of experts determined the criteria for drug authorization according to evidence-based knowledge accumulated in the literature, and an ophthalmic specialist team approved or rejected applications submitted by fellow ophthalmologists**.** For first-time funding requests, the ophthalmic specialist team determined whether a patient who applied for funding of ranibizumab failed to respond to first-line bevacizumab, and matched the criteria for switching the treatment. For continuation of funding requests, treating ophthalmologists were required to prove that ranibizumab was effective or superior to previous bevacizumab treatment for the individual patient.

Our analysis showed that from the approximately 18,000 bevacizumab-treated members of Clalit Mushlam, 5642 (31%) requested funding of second-line treatment with ranibizumab owing to lack of sufficient improvement or an adverse reaction to bevacizumab. Approval was ultimately granted to 95% of the patients. A similar overall rejection rate of 4.4% was described in an earlier analysis of a prior authorization program for non-ophthalmic medications in a Medicaid HMO [3].

Another study described the prior-authorization program for cyclooxygenase-2 inhibitors relative to nonselective nonsteroidal anti-inflammatory drugs, which resulted in a decrease in prescription rate of the costlier drug [4]. However, in their restriction of cyclooxygenase-2 inhibitors, not all the programs considered its lower rates of gastrointestinal complications or other evidence-based criteria. Thus, in the absence of individual patient data, the clinical appropriateness of the prior authorization programs could not be determined [27].

To the best of our knowledge, gatekeeping rests mainly on the shoulders of primary care physicians. This is because this role is unparented, even in the sensitive field of retina care. Given that primary physician gatekeeping can reduce healthcare costs by a reported 15–26% [28, 29], we might expect a similar decrease in cases where peers within the same medical field authorize procedures and medications according to pre-set clinical criteria.

The clinical criteria for the approval of ranibizumab use under Clalit Mushlam were continuously updated during the study period. The prospective accumulation of such a large body of individual patient data is invaluable for future assessments of the clinical benefit of second-line ranibizumab in patients with retinal disease, in addition to the clinical benefit of a prior-authorization policy itself. Although several studies have investigated the economic savings and reduction in medication use following prior authorization in Medicaid programs [4, 30–33], none addressed their clinical benefit yet. The requirements for clinical appropriateness varied among the approval procedures of the different states, and none of the studies had access to patient data.

The high approval rate of requests (94.3%) together with a relatively low (31%) percentage of requests for funding of second-line treatment might be due a “sentinel effect” to accurate adaptation of the guidelines for ranibizumab treatment and careful selection of cases for submission by the treating ophthalmologists, knowing that their requests are going to be inspected by their peers.

The reason for the approval rate of 84% for the first-time requests, compared to the approval rate of 76% for continuation requests, might be associated with the higher adherence rate of physicians to their prior recommendations despite absence of benefit evidence, hoping for improvement. This might be due to the higher threshold of criteria for ranibizumab treatment continuation, rather than initiation. These should be further investigated.

In our cohort, 99.8% of the patients approved for ranibizumab treatment received the injections. This finding suggests that the co-payment did not deter treatment and emphasizes the necessity of the treatment in the opinion of these patients.

The number of 41,739 ranibizumab injections that were supplied to the retina units during the research period was higher than the expected 39,834 injections (13,278 approved funding requests x three injections per approval). This was mainly because requests for two eyes of the same patient were submitted on the same application form, and thus were counted as one approved request.

Eventually the annual number of injected ranibizumab vials was 10,888, while the estimated number expected by the steering committee was 16,000 vials. The gap may be due to several reasons: (i) the estimation by the steering committee may have been overly cautious; (ii) intervals between injections may have increased due to stable improvement of the retinal state; and (iii) the retina units may have had a limited capability to treat such a large quantity of patients.

We believe that the inclusion of funding of such a novel treatment as ranibizumab (without prior authorization) to Clalit Mushlam could have potentially ended in a switch of treatment in the vast majority of the approximately 18,000 bevacizumab-treated members of Clalit Mushlam. Hypothetically, taking into consideration both a $1000 difference per injection between the costlier ranibizumab and bevacizumab, and 900 preventions of patient treatment switching (5% of 18,000 patients), about 2.5 million dollars were potentially saved within the first 6 months after the rejection and potentially more thereafter. This resulted in an overall actual cost of $130,000 for the entire 45-month period of the study, and without substantially affecting patient outcomes. Similar assumptions and savings of the forecast model were predicted in switching to a less expensive “blindness drug” [34]. Additionally, the possibility that successful outcomes of treatment may cause an increase of incoming requests should be taken into account.

It is important to note and understand that two parallel processes are working simultaneously when establishing guidelines and regulations. The first is establishing and formulating a structured set of rules and evidence-based guidelines to be used by all physicians (retina specialists in our case) when advancing the treatment from first line to second line of treatment and conveying the expectations to keep those guidelines. The second reason is to actively control and enforce those guidelines. While such control is visibly applied and maintained though time, a measure of self-control of self “censorship” by physician is formed and the system is decreasingly challenged. This point was clearly demonstrated by the fact that only a third of bevacizumab treated patients applied to switch to ranibizumab. Both processes (active control and passive indirect control) reduce cost and are mutually complimenting and dependent for success.

This paper depicts a novel approach to cost containment, describing a peer review system based on trust in accuracy of the data and cooperation between the physician and regulatory agency. In this case, such a system facilitated formation of a consensus regarding treatment parameters, and the constant monitoring resulted in strict self-censorship which allowed for minimal formal rejections while containing the expenses within target values.

It is our belief that regulatory agencies be it policy makers, hospital directors, or health management professionals may benefit from implementing this approach or a variation of it into their regulatory schemes, thus allowing for best individualized treatment for each patient, while keeping within the budget, and with optimal interaction between the treating physician and the regulatory agencies.

A limitation of the present report is the non-uniform dispersion of indications for ranibizumab treatment throughout the study period. The changing available literature, pointing to new avenues of treatments, points to the need for flexibility. Second, we relied on the Clalit Health Services report which rounded, and may have miscounted, the number of bevacizumab-treated patients. Further study is needed to assess the health outcome of this program and physicians and patients satisfaction compared to the other budget regulatory option. Additionally, future studies may focus on detecting physicians with high rates of use of the more expensive medication comparing to physicians with low rates. Such a differentiation may help in simplifying the process for physicians with low rates of use of this medication by waiving the need for them to submit an authorization request. This will shorten the process on one hand but may harm the uniformity of the national process on the other hand.

## Conclusions

We describe a prior authorization program for a costly and effective medication for retinal diseases, as a replacement for a cheaper treatment, in case of the latter’s failure. This was enabled by a careful selection of patients by ophthalmologists’ determined criteria for the funding of an almost 10-times costlier treatment. This approach enabled treatment for thousands of patients under professionally accepted clinical considerations, within a framework budget. The data presented in this study show a relatively low request rate for funding with a high approval rate, proving physician not to recommend treatment unless the treating specialist truly believes the patient requires it and the formation of a successful sentinel effect.

Lastly, owing to the clinical considerations that exist in this process, an integral part to assess is not only its economic, but also its clinical benefits. We aim to continue collecting data and investigating the clinical aspects of this intervention.

## Data Availability

This research was presented in a free paper session at the annual meeting of the Israeli Ophthalmology Society, Tel-Aviv, Israel, June 6–7, 2017.
